# HDAC inhibitor ITF2357 reduces resistance of mutant-KRAS non-small cell lung cancer to pemetrexed through a HDAC2/miR-130a-3p-dependent mechanism

**DOI:** 10.1186/s12967-023-03973-3

**Published:** 2023-02-15

**Authors:** Jian Cui, Fei Xu, Wei Bai, Tiantian Zhao, Junbo Hong, Wei Zuo

**Affiliations:** 1 Department of Respiratory and Critical Care Medicine, The First Affiliated Hospital of Nanchang University, No. 17, Yongwaizheng Street, Nanchang, 330006 Jiangxi People’s Republic of China; 2 Jiangxi Institute of Translational Medicine, The First Affiliated Hospital of Nanchang University, Nanchang, 330006 People’s Republic of China; 3 Department of Gastroenterology, The First Affiliated Hospital of Nanchang University, Nanchang, 330006 People’s Republic of China

**Keywords:** Histone deacetylases inhibitor ITF2357, HDAC2, MicroRNA-130a-3p, Rad51, KARS, Mutant-KRAS non-small cell lung cancer, Pemetrexed, Chemoresistance

## Abstract

**Background:**

Histone deacetylases (HDAC) contribute to oncogenic program, pointing to their inhibitors as a potential strategy against cancers. We, thus, studied the mechanism of HDAC inhibitor ITF2357 in resistance of mutant (mut)-KRAS non-small cell lung cancer (NSCLC) to pemetrexed (Pem).

**Methods:**

We first determined the expression of NSCLC tumorigenesis-related HDAC2 and Rad51 in NSCLC tissues and cells. Next, we illustrated the effect of ITF2357 on the Pem resistance in wild type-KARS NSCLC cell line H1299, mut-KARS NSCLC cell line A549 and Pem-resistant mut-KARS cell line A549R in vitro and in xenografts of nude mice in vivo.

**Results:**

Expression of HDAC2 and Rad51 was upregulated in NSCLC tissues and cells. Accordingly, it was revealed that ITF2357 downregulated HDAC2 expression to diminish the resistance of H1299, A549 and A549R cells to Pem. HDAC2 bound to miR-130a-3p to upregulate its target gene Rad51. The in vitro findings were reproduced in vivo, where ITF2357 inhibited the HDAC2/miR-130a-3p/Rad51 axis to reduce the resistance of mut-KRAS NSCLC to Pem.

**Conclusion:**

Taken together, HDAC inhibitor ITF2357 restores miR-130a-3p expression by inhibiting HDAC2, thereby repressing Rad51 and ultimately diminishing resistance of mut-KRAS NSCLC to Pem. Our findings suggested HDAC inhibitor ITF2357 as a promising adjuvant strategy to enhance the sensitivity of mut-KRAS NSCLC to Pem.

**Supplementary Information:**

The online version contains supplementary material available at 10.1186/s12967-023-03973-3.

## Background

Non-small-cell lung cancer (NSCLC) accounts for approximately 85% of the total lung cancer cases and represents a major reason for cancer-related deaths [1]. It is known that the molecular epidemiology of NSCLC involves druggable genetic alterations such as the mutations of EGFR, KRAS, BRAF, MET, and HER2 [2]. At present, a low radiation dose chest CT scan is recommended as a golden screening method of lung cancer for screen eligible population [3]. However, unfortunately, NSCLC is usually diagnosed at the metastatic phase, and the median survival is only 1 year [4]. To our acknowledge, pemetrexed (Pem), a type of folate analogue metabolic inhibitor used for mammalian cells, can be toxic to cancer cells through interference with their new biosynthesis of nucleotides to induce cell apoptosis and has been approved to be used for patients with NSCLC [5].

Histone deacetylase (HDAC) inhibitors are representative of an extensive series of targeted anti-tumor agents, among which some have been approved for clinical treatment of cancers including givinostat (ITF2357) [6]. Intriguingly, it has been reported that ITF2357 can aid in enhancement of the cytotoxicity of Pem via induction of apoptosis as well as autophagy in NSCLC [7]. Of note, overexpressed HDAC2 could lead to reduced cisplatin sensitivity of NSCLC cells induced by valproic acid [8]. Rad51, identified as the key protein in the homologous recombination pathway, shares great association with therapy resistance and genomic stability [9]. Of note, decreased expression of Rad51 due to astaxanthin could result in promotion of mitomycin C-induced cytotoxicity in human NSCLC cells [10].

Interestingly, our bioinformatic analyses suggested that MicroRNA (miR)-130a-3p, retrieved from a NSCLC-related microarray dataset, could target Rad51. MiRs, small single stranded non-coding RNA molecules with the ability of gene regulation at the posttranscriptional level, have been highlighted to play an important role in NSCLC [11]. A previous study has found that miR-130a-3p, was notably downregulated in patients with NSCLC relative to that in healthy individuals [12]. Moreover, a prior study has pointed out the suppression of HDAC3 on miR-130a-3p in breast cancer, which led to augmented escape of cancer cells from immune surveillance [13]. Considering all the above findings, we hence hypothesized in the current study that HDAC inhibitor ITF2357 was likely to regulate the chemoresistance of mutant (mut)-KRAS NSCLC to Pem, with the involvement of the miR-130a-3p/HDAC2/Rad51 axis.

## Methods

### Ethics statement

The study protocol was in line with the ethical guidelines of the Helsinki Declaration and approved by Institutional Ethics Review Committee of The First Affiliated Hospital of Nanchang University. The animal experiments in this study were conducted under the *Guide for the Care and Use of Laboratory Animals* published by the National Institutes of Health.

### Clinical sample collection

Sixty patients diagnosed with stage I, II or IIIa NSCLC who underwent resection surgery in The First Affiliated Hospital of Nanchang University from January 2016 to December 2017 were enrolled in this study. Patients with a recent history of other cancers, or with recurrent or primary NSCLC, or who had received chemotherapy or radiotherapy before surgery were excluded. Participants had complete clinical and pathological follow-up data. Paired NSCLC and adjacent normal tissues obtained from the 60 patients were immediately frozen in liquid nitrogen for RNA and protein extraction. Sixty collected NSCLC tissue samples were made into formalin-fixed and paraffin-embedded NSCLC specimens to detect the protein expression of HDAC2 by immunohistochemistry. Follow-up monitoring of the 60 patients were conducted to record their survival.

### Cell culture

Human lung cancer cell lines, namely, mut-KARS A549 cell line and wild-type (wt) H1299 cell line, and human embryonic kidney cell line 293 T were purchased from Shanghai Institute of Biological Sciences, Chinese Academy of Sciences (Shanghai, China). The human lung cell line BEAS-2B was purchased from ATCC (Manassas, VA). The cells were cultured in DMEM medium (Gibco, Carlsbad, CA) supplemented with 10% heat-inactivated FBS, penicillin (100 U/mL) and streptomycin (100 μg/mL) in a CO_2_ incubator with 5% humidity at 37℃.

A549 cells were repeatedly exposed to a single high concentration of Pem (Pem inhibition concentration is IC50). Pem was purchased from Eli Lilly and Company (Indianapolis, IN; A762406C). Pem-treated cells were then washed with PBS and cultured in fresh medium without Pem. Following that, surviving cells were cultured. A549 cells exposed to Pem recovered and showed logarithmic growth after 2 weeks. Cells growing exponentially were trypsinized and exposed to Pem again for 48 h, the resistance of which was detected discontinuously until the experimental requirements were met. After 5 months, a Pem-resistant cell line was established and named A549R. The A549R cells were cultured (5% CO_2_, 37 °) for 1 month and passaged. The resistance of A549R cell line was confirmed again. Finally, A549R cells were cultured in Pem-free medium for another month.

### Construction of stably transduced cell line

After construction of lentiviral vectors carrying oe-NC and oe-Rad51, stable genetic A549 cell lines were obtained. The lentiviral vector was pLV-EGFP-N (overexpression vector, oe-), which was purchased from GenePharma (Shanghai, China). 293T cells were cultured in RPMI-1640 complete medium, and passaged every other day. The virus was collected, and then was added into A549R cells (1 × 10^8^ TU/mL) for infection to obtain stably transduced cell lines.

miR-130a-3p mimic: 5'-CAGTGCAATGTTAAAAGGGCAT-3' (sense); mimic NC: 5'-GGTAACAATATCGGGTCAAGAT-3' (sense).

### RNA quantification

Trizol reagent (Invitrogen, Carlsbad, CA) and PrimeScript RT Master Mix (Takara Bio Inc., Otsu, Shiga, Japan) were utilized to extract total RNA from cells and tissues. Then, mRNA was reversely transcribed into cDNA; A Poly(A) tailing reverse transcription kit (B532451-0010, Sangon, Shanghai, China) was utilized to reversely transcribed miRNA into cDNA. All primers were synthesized by GenePharma, as listed in Additional file 2: Table S1. β-actin was utilized as an internal reference for mRNA normalization and U6 was used for miR normalization. 2^−ΔΔCt^ method was employed to quantify the relative expression level of target genes.

### Western blot assay

Tissue or cells were lysed with high-efficiency radioimmunoprecipitation assay (RIPA) lysis buffer (R0010, Solarbio, Beijing, China) to extract total protein, followed by measurement of protein concentration utilizing BCA kits (20201ES76, Yeasen, Shanghai, China). With the protein concentration adjusted to 1 μg/μL, the protein lysate was directly loaded to 10–12% SDS–polyacrylamide gel electrophoresis and transferred onto a PVDF membrane to carry out western blot (0.2 μM, Bio-Rad Laboratories, Hercules, CA). Cells were sealed with 5% skim milk for 1 h, and then incubated with the primary antibodies (Rad51; 1:500, sc-398587, Santa Cruz Biotechnology, Santa Cruz, CA), HDAC2 (1:1000; 5113, Cell Signaling Technology, Beverly, MA), and β-actin (1:2000, sc-8432, Santa Cruz Biotechnology) at 4 ℃ overnight. Afterwards, the membrane was incubated for 1 h with horseradish peroxidase (HRP)-labeled goat anti-rabbit against IgG (ab205718, 1:2000, Abcam, Cambridge, MA) or goat anti-mouse diluent (ab6789, 1:5000, Abcam) at room temperature. Immobilon Western chemiluminescence HRP substrate (Millipore, Billerica, MA) and Bio-Rad Chemi Doc MP were used to observe Western blots. Total protein and cytoplasmic protein were determined using β-actin as internal reference, and the ratio of gray value of target band and internal reference band indicated relative protein expression level, as analyzed by ImageJ 6.0 software (National Institutes of Health, Bethesda, MD).

### MTT assay

NSCLC cells were seeded in a 96-well plate (3 × 10^4^ cells/well), and cell viability was measured by MTT assay. Cells were treated with different concentrations of HDAC inhibitor ITF2357 or Pem. After incubation for 24 h, 10 μL MTT solution (5 mg/mL, GD-Y1317, Guduo Biotechnology, Shanghai, China) was added to each well of the 96-well plate, and then incubated in an incubator for 4 h, and the supernatant was subsequently discarded. Then, 100 μL dimethyl sulfoxide was added to each well to make methyl Zan crystal dissolve completely. The 96-well plate was placed in a microplate reader, and the absorbance at 570 nm and 630 nm were measured.

### Transwell assay

Matrigel was used for invasion detection but not for migration assay. (Corelle, New York). Matrigel was added to the apical chamber of the 24-well Transwell plate (8 μm pore size), which was incubated in an incubator at 4℃ for 30 min to polymerize Matrigel into gel. Basement membrane hydration was performed before use. NSCLC cells were cultured in serum-free medium for 12 h. The cells were harvested and re-suspended in serum-free medium (10^5^ cells) and seeded in the apical chamber. NSCLC cells (2 × 10^4^ cells/well) were seeded in the basolateral chamber of the Transwell plate. After 24 h of incubation at 37 ℃, the invasive cells were stained with crystal violet for 15 min and photographed under an inverted light microscope (Carl Zeiss MicroImaging, Inc., Thornwood, NY). The invasive and migratory abilities of NSCLC cells were counted and analyzed by imagine J software.

### Radioimmunoprecipitation (RIP)

RIP Kits (17-701, Millipore) were used to detect the binding of Rad51 to Ago2 protein according to the instructions of the kits. When the cell confluence reached 80–90%, the medium was discarded. Cells were lysed with RIPA lysis buffer (P0013B, Beyotime, Shanghai, China) for 5 min, and centrifuged at 4 ℃ for 10 min at 14,000 rpm, with the supernatant subsequently obtained. One part of the cell extract was taken out as input, and the other part was incubated with antibody for coprecipitation. The specific steps were as follows: 50 μl magnetic beads were washed and then resuspended in 100 μL RIP wash buffer, and 5 μg antibody was added according to the experimental grouping for binding purpose. The magnetic bead-antibody complex was resuspended in 900 μL RIP wave buffer and incubated with 100 μL cell extract at 4 ℃ overnight.. The complex was then harvested, and samples and inputs were detached with proteinase K to extract RNA for subsequent qPCR detection of Rad51. Rabbit anti-mouse against Ago2 (1:100, ab32381, Abcam) and goat anti-mouse against IgG (1:100, ab205719, Abcam) were as negative control (NC).

### RNA pull-down assay

The cells were transfected with biotin-labeled Bio-NC, Bio-Rad51-wt and Bio-Rad51-mut RNA (50 nM each). After 48 h of transfection, the cells were collected and washed with PBS. Then, the cells were incubated with specific cell lysate (20164, Thermo Fisher Scientific Inc., Waltham, MA) for 10 min. After that, 50 mL sample cell lysate was separately packed. The residual lysate was incubated at 4℃ for 3 h with M-280 streptavidin magnetic beads pre-coated with RNase-free and yeast tRNA (R8759, Sigma-Aldrich, St Louis MO), followed by two rinses with cold lysate, three rinses with low-salt buffer, and one rinse with high-salt buffer. The binding RNA was purified by Trizol, and then the enrichment of miR-130a-3p was detected by RT-qPCR.

### Dual luciferase reporter gene assay

The targeting relationship between miR-130a-3p and Rad51 was verified using biological prediction website and luciferase reporter gene assay. The binding sites of miR-145 and Rad51 were analyzed, and the fragment sequence containing the action site was obtained. The 3’UTR region of Rad51 and the sequences after site-directed mutagenesis of miR-130a-3p binding site were cloned into the target sequences of psiCheck2 plasmid downstream of luciferase reporter gene (Hanbio Biotechnology, Shanghai, China). They were named Rad51-mut and Rad51-wt respectively. The luciferase activity was measured using luciferase assay kits (Promega, Madison, WI). After incubation for 48 h, the cells were lysed and the luciferase activity of firefly was measured using the dual luciferase system (Promega).

### Chromatin immunoprecipitation (ChIP)

ChIP assay kits (Millipore) were used as follows: A total of 1 × 10^7^ cells were fixed with 1% formalin for 10 min to make DNA and protein cross-linked. After that, the cells were subjected to SDS lysis, and the DNA was randomly sonicated into 500–1000 bp fragments. After 12,000 *g* centrifugation at 4 ℃, the supernatant was collected and placed into two tubes, which were respectively mixed with the specific antibodies of the target proteins. Pol II (05–623, 10 mL/mg total protein, Millipore), AcH4 (06-866, 10 mL/mg total protein, Millipore), HDAC2 (17-10237, 5 mL/mg total protein, Millipore) and IgG in the control group. For AcH4 ChIP, sodium butyrate (20 mM, 19-137, Millipore) was added to all solutions to maintain histone acetylation. ChIP DNA was purified and eluted with 100 μL H_2_O, and 2.5 μL ChIP DNA was extracted for qPCR detection. Primers: RNA Pol II: 5ʹ-CAGGGACTGGGAGAAGGA-3ʹ (forward), 5ʹ-CACTGCTAGTGACAGGTGCA-3ʹ (reverse); AcH4 and HDAC2: 5ʹ-GCCCCATCCCCTGCTGCT-3ʹ (forward), 5ʹ-CAGGCCCAGCGACTCACC-3ʹ (reverse).

### Flow cytometry

After 48 h of transfection, cells were detached with 0.25% trypsin (without EDTA) and collected, followed by centrifugation and removal of the supernatant. Based on the instructions of Annexin-V-fluorescein isothiocyanate (FITC) cell apoptosis detection kit (556547, Shanghai Shuojia Biotechnology Co., Ltd., Shanghai, China), Annexin-V-FITC, PI and HEPES buffer solution were prepared into Annexin-V-FITC/PI staining solution at the ratio of 1:2:50. Next, per 100 μL of dye solution was used to resuspend 1 × 10^6^ cells, followed by 15-min incubation with 1 mL HEPES buffer solution. The 515 nm and 620 nm band-pass filters were stimulated at 488 nm wavelength to detect FITC and PI fluorescence.

### Xenografts in nude mice

Female athymic nude mice (Envigo, Shanghai, China; 5–6 weeks old) were raised in a SPF animal room (25 ℃, 70% humidity) under 12-h light/dark alternation. About 5 × 10^6^ H1299, A549 or A549R cells were suspended in 100 μL PBS containing 20% Matrigel and injected subcutaneously into the right flank of each mouse. The tumor size was measured weekly with a digital caliper and calculated by the following formula: tumor size = a × b^2^/2, where a represents the larger size and b represents the smaller size. When the average tumor volume of mice was about 100 mm^3^, A549R tumor-bearing mice were randomly into four groups (5 in per group) and accordingly injected with (1) PBS, (2) ITF2357 (HDAC inhibitor, 50 mg/kg), (3) Pem (100 mg/kg) and (4) ITF2357 + Pem (sequential injection of Pem and then of ITF2357) every other day via tail vein. The injection volume of each was 200 μL. The tumor volume was closely monitored with time. After 20 days, the mice were euthanized, and tumor xenografts were collected, weighed and analyzed.

### TUNEL staining

The experiment was carried out following the protocols of apoptosis detection kit (40306ES60/40308ES60, Yeasen Company). The slices were treated with 20% normal bovine serum for 30 min at room temperature, and then incubated with 50 μL of TUNEL reaction mixture at 37 ℃ for 90 min (negative pairs were not added with TUNEL reaction mixture). Next, the slices were treated with 3% H_2_O_2_ methanol solution for 10 min at room temperature, and then incubated with horseradish peroxidase (POD) solution (50 μL/tablet) at 37 ℃ for 30 min. Following diaminobenzidine/hydrogen peroxide (DAB/H_2_O_2_) color development, the slices were stained with hematoxylin and then photographed under a positive fluorescence microscope (BX63, Olympus, Tokyo, Japan).

### Immunohistochemistry and in situ hybridization

The formaldehyde fixed (4%) tumor tissues were embedded in paraffin. After being baked, the samples were successively placed in xylene solution, and then soaked for 15 min after xylene was replaced. Then the samples were dehydrated with gradient ethanol. Finally, the samples were washed with double distilled water. Each slice was dipped with 3% H_2_O_2_ to block endogenous peroxidase. After addition of citric acid buffer, the samples were boiled in a microwave oven for 3 min, treated with antigen repair solution, and allowed to rest at room temperature for 10 min. Next, normal goat serum blocking solution (Sangon, Shanghai, China) was used to incubate the samples at room temperature for 30 min. Diluted primary antibodies against HDAC2 (1:200, ab32117, Abcam) and Rad51 (1:500, ab133534, Abcam) were dropped to the samples for incubation at 4 ℃ overnight. The following day, the secondary antibody goat anti-rabbit against IgG (ab6721, 1:5000, Abcam) was used to incubate the samples for 30 min. Following incubation with SABC (Vector Company) in a 37 ℃ incubator for 30 min, DAB reagent kit (P0203, Beyotime). The samples were dyed in hematoxylin and sealed with neutral resin, and observed under an upright microscope (BX63, Olympus).

According to the instruction of in situ hybridization detection kit (Bio-high technology, Shijiang Huang, China), digoxigenin-labeled oligonucleotide probes (5ʹ-GCCCTTTTACATGCACTG-3ʹ) were used to detect the presence of miR-130a-3p in tumor tissues.

### Hematoxylin and eosin (HE) staining

Complete tumor tissue sections were fixed at room temperature for 30 s, and stained with hematoxylin (60 ℃) for 60 s. Following a rinse with 1% hydrochloric acid alcohol differentiation solution, sections were counterstained with eosin for 3 min, dehydrated, and cleared with xylene. After that, sections were cleared, sealed, and observed under a microscope (BX63, Olympus).

### Statistical analysis

All data i were analyzed utilizing the SPSS 21.0 statistical software (IBM, Armonk, NY). Each experiment was repeated three times. The measurement data were expressed by mean ± standard deviation. Paired *t* test was used to compare data between NSCLC tissue and adjacent normal tissue. Data between two groups were compared by unpaired *t* test, and those among multiple groups by one-way analysis of variance (ANOVA), followed by Tukey’s post hoc tests. Data among multiple groups at different time points were compared by repeated measures ANOVA, followed by Bonferroni post hoc tests. Kaplan–Meier method was used to calculate the survival rate of patients. *p* < 0.05 was indicative of statistically significant difference.

## Results

### HDAC2 expression was upregulated in NSCLC tissues and cells

**Fig. 1:**
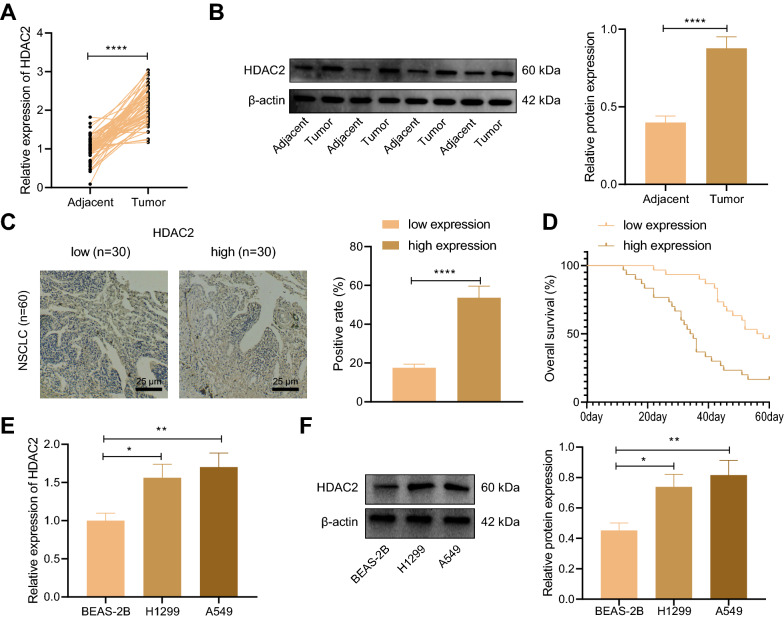
HDAC2 expression is upregulated in NSCLC tissues and cells. **A** RT-qPCR was used to detect the mRNA expression of HDAC2 in NSCLC tissues and adjacent normal tissues of NSCLC patients. **B** Western blot was used to detect the protein expression of HDAC2 in NSCLC tissues and adjacent normal tissues of NSCLC patients. **C** Immunohistochemistry was used to detect the expression of HDAC2 in NSCLC tissues of NSCLC patients. **D** Survival analysis of NSCLC patients (NSCLC patients were divided into high/low HDAC2 expression groups according to the median value of the HDAC2 expression level). **E** RT-qPCR was used to detect the mRNA expression of HDAC2 in a human lung cell line BEAS-2B, a wild type-KARS NSCLC cell line H1299, and a mut-KARS NSCLC cell line A549. **F** Western blot was used to detect the protein expression of HDAC2 in a human lung cell line BEAS-2B, a wild type-KARS NSCLC cell line H1299, and a mut-KARS NSCLC cell line A549. *p < 0.05. **p < 0.01. ****p < 0.0001. Paired *t* test was used to compare data between NSCLC tissue and adjacent tissue. Data between two groups were compared by unpaired *t* test, and those among multiple groups by one-way ANOVA, followed by Tukey’s post hoc tests. Kaplan–Meier method was used to calculate the survival rate of patients. Cellular experiments were repeated three times

Initially, we determined HDAC2 expression level in lung tissues of enrolled NSCLC patients. RT-qPCR and Western blot results displayed that HDAC2 expression was up-regulated in NSCLC tissues relative to adjacent normal tissues (Fig. 1A, B). In addition, the NSCLC patients were divided into high/low HDAC2 expression groups according to the median value of the HDAC2 expression level in NSCLC tissues of all patients, and the overall survival time of the low HDAC2 expression group was found to be notably longer than that of the high HDAC2 expression group (Fig. 1C, D). Subsequently, we determined HDAC2 expression in human normal lung cell line BEAS-2B, KRAS WT NSCLC cell line H1299 and mut-KRAS NSCLC cell line A549. The results displayed that HDAC2 expression in H1299 and A549 cells was higher than that in BEAS-2B cells (Fig. 1E, F). These results demonstrated upregulated HDAC2 expression in NSCLC tissues and cells.

### HDAC inhibitor ITF2357 inhibited the expression of HDAC2 and enhanced the chemotherapeutic effect of Pem on NSCLC

**Fig. 2:**
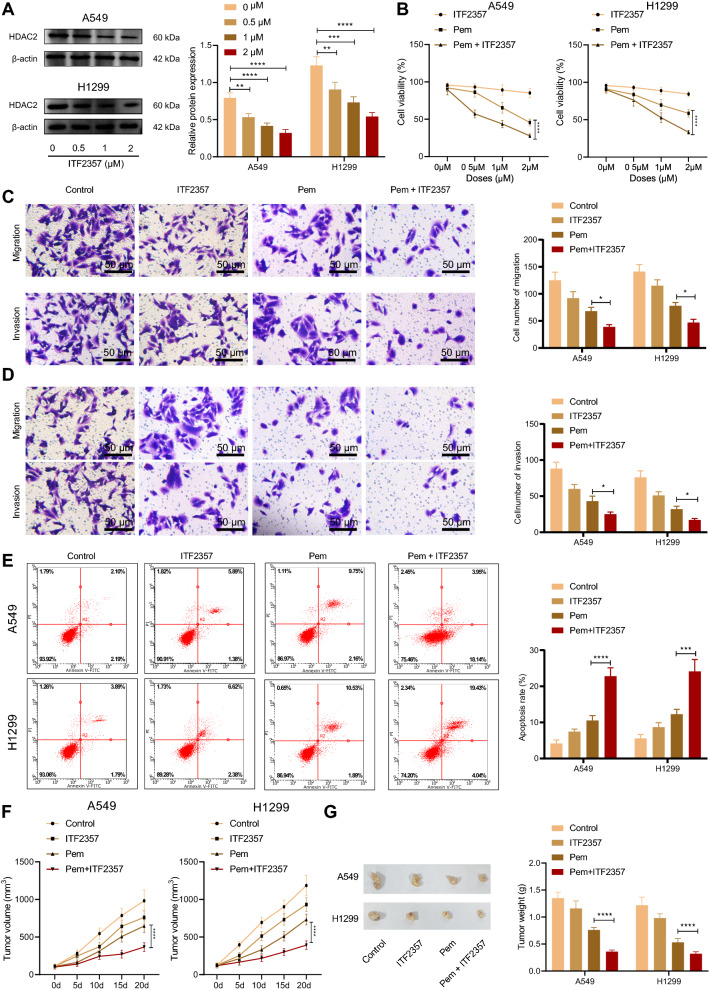
HDAC inhibitor ITF2357 inhibits the expression of HDAC2 and enhances the chemotherapeutic effect of Pem on NSCLC. **A** The protein expression of HDAC2 in a wild type-KARS NSCLC cell line H1299 and a mut-KARS NSCLC cell line A549 was detected by Western blot after different treatment. **B** The viability of cells was detected by MTT assay at different concentrations of ITF2357 or Pem. **C** The migration of cells upon different treatments was detected by Transwell assay. **D** The invasion of cells upon different treatments was detected by Transwell assay. **E** The apoptosis of cells upon different treatments was detected by flow cytometry. **F** The change of tumor size in mice upon different treatments (n = 5). **G** The weight analysis of tumors in mice upon different treatments (n = 5). **p < 0.05. ***p < 0.01. ****p < 0.001. Data among multiple groups were compared by one-way ANOVA, followed by Tukey’s post hoc tests. Data among multiple groups at different time points were compared by repeated measures ANOVA, followed by Bonferroni post hoc tests. Cellular experiments were repeated three times

Next, we selected HDAC inhibitor ITF2357 to verify whether inhibition of HDAC2 enhanced the chemotherapeutic effect of Pem on NSCLC. First, Western blot analysis displayed showed that the protein expression level of HDAC2 in A549 and H1299 cells was decreasing following the increasing concentration of ITF2357 (Fig. 2A). Subsequently, MTT assay revealed that revealed that ITF2357 treatment itself showed no obvious effects on cell viability, whereas the combination of ITF2357 and Pem resulted in much more greater inhibiting effects, relative to Pem alone, on the viability of H1299 and A549 cells(Fig. 2B). Similarly, ITF2357 treatment itself showed no obvious effects on NSCLC cell invasion, migration, and apoptosis, whereas it could notably elevate the inhibiting effect of Pem on the invasive and migratory potentials of NSCLC cells, as reflected by the Transwell assay (Fig. 2C, D) as well as the stimulating effect of Pem on NSCLC cell apoptosis, as reflected by glow cytometry (Fig. 2E).

Further to verify the synergistic effect of ITF2357 and Pem on in vivo tumor growth, tumor-bearing mice were established by subcutaneous injection of H1299 and A549 cells respectively, and ITF2357 alone or Pem in combination were given. The results displayed that in the H1299 and A549 tumor-bearing mice, compared with those upon Pem treatment alone, the tumor volume and tumor weight in the presence of of Pem + ITF2357 were markedly decreased, and ITF2357 alone exhibited no obvious effect on tumor volume and weight (Fig. 2F, G). In conclusion, the HDAC inhibitor ITF2357 inhibited the expression of HDAC2 and enhanced the chemotherapeutic effect of Pem on NSCLC.

### HDAC inhibitor ITF2357 reduced the resistance of mut-KRAS NSCLC cells to Pem

Based on the fact that ITF2357 could enhance the efficacy of Pem to mut-KARS and wt-KARS NSCLC cells, we further studied whether ITF2357 reduced the resistance of mut-KRAS NSCLC cells to Pem.

**Fig. 3:**
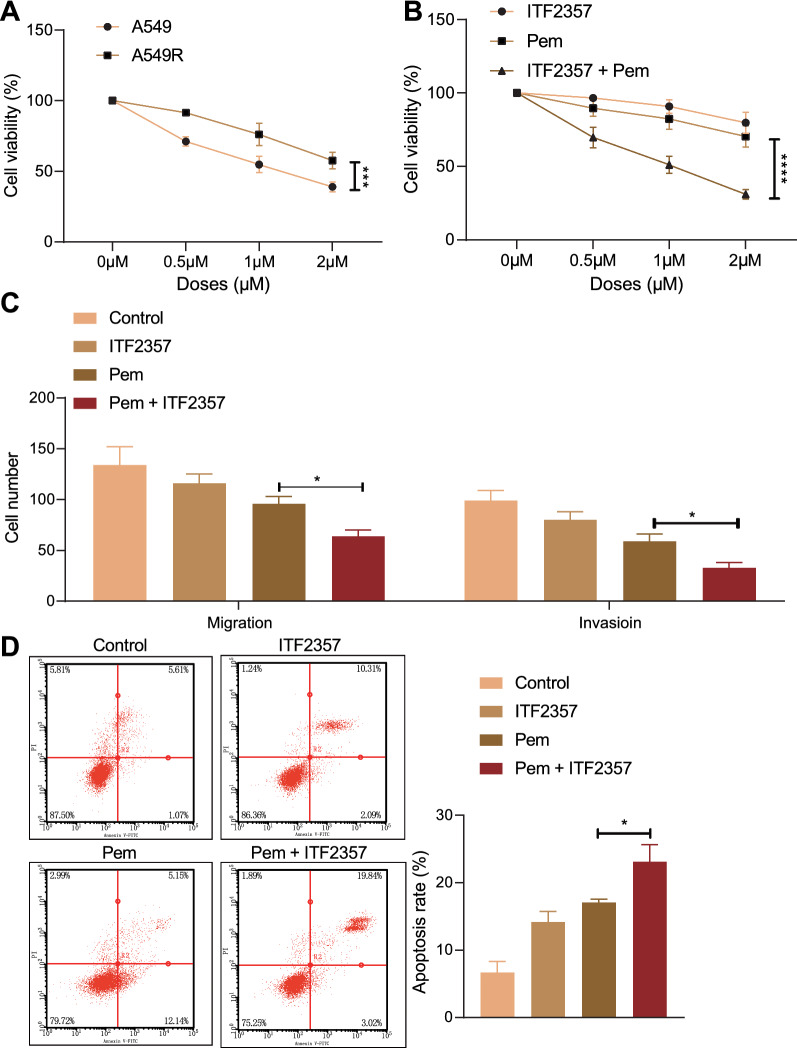
HDAC inhibitor ITF2357 reduces the resistance of mut-KRAS NSCLC cells to Pem. **A** MTT assay was used to detect the viability of cells (A549, A549R) upon different treatments at different concentrations of Pem. **B** MTT assay was used to detect the viability of cells upon different treatments at different concentrations of ITF2357. **C** Transwell assay was used to detect the migration and invasion of cells upon different treatments (ITF2357 or Pem treatment alone, or their combination). **D** Flow cytometry was used to detect the apoptosis of cells upon different treatments (ITF2357 or Pem treatment alone, or their combination). *p < 0.05. **p < 0.01. ****p < 0.0001. Data among multiple groups were compared by one-way ANOVA, followed by Tukey’s post hoc tests. Data among multiple groups at different time points were compared by repeated measures ANOVA, followed by Bonferroni post hoc tests. Cellular experiments were repeated three times

Firstly, we established the Pem-resistant A549R cells. As shown in Fig. 3A, MTT results showed that relative to that of the A549 cells, the survival rate of A549R cells was increased at the same dose of Pem, which proved that the Pem-resistant A549 cells were successfully established. Subsequently, further MTT results unveiled that the survival rate of A549R cells treated with ITF2357 + Pem was notably lower than that of A549R cells exposed to Pem alone (Fig. 3B). In addition, Transwell assay revealed that relative to Pem alone, Pem + ITF2357 notably decreased the invasive and migratory potentials of A549R cells (Fig. 3C). Flow cytometry demonstrated that compared with Pem alone, Pem + ITF2357 increased the apoptosis of A549R cells (Fig. 3D). In conclusion, ITF2357 reduced the resistance of mut-KRAS NSCLC cells to Pem.

### HDAC inhibitor ITF2357 reduced the resistance of mut-KRAS NSCLC cells to Pem by inhibiting HDAC2/Rad51

It has recently been reported that KRAS mutant can promote Rad51 expression to increase the chemotherapy resistance of cancer cells, while inhibiting HDAC can down-regulate Rad51 to counteract Rad51-mediated resistance of cancer cells [14, 15].

**Fig. 4:**
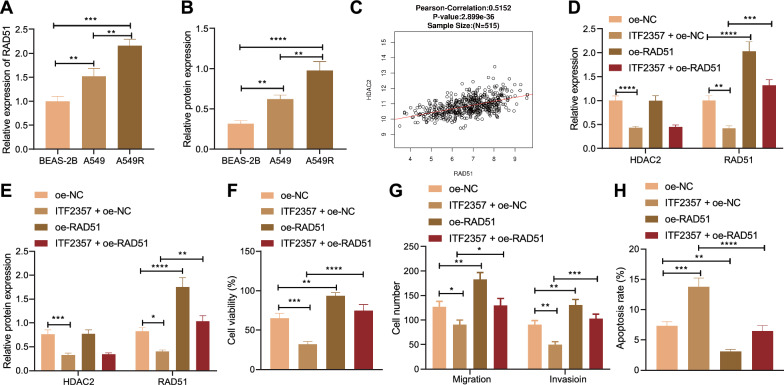
HDAC inhibitor ITF2357 reduces the resistance of mut-KRAS NSCLC cells to Pem by inhibiting HDAC2/Rad51. **A** RT-qPCR was used to detect the gene expression level of Rad51 in cells upon different treatments. **B** Western blot was used to detect the protein expression level of Rad51 in cells upon different treatments. **C** LinkedOmics database was used to analyze the correlation between Rad51 and HDAC2 in TCGA-LUAD. **D** RT-qPCR was used to detect the gene expression levels of HDAC2 and Rad51 in cells upon different treatments. **E** Western blot was used to detect the protein expression level of Rad51 in cells upon different treatments. **F** The viability of cells upon different treatments was detected by MTT. **G** The migration and invasion of cells upon different treatments were detected by Transwell assay. **H** The apoptosis of cells upon different treatments was detected by flow cytometry. *p < 0.05. **p < 0.01. ***p < 0.001. ****p < 0.0001. Data among multiple groups were compared by one-way ANOVA, followed by Tukey’s post hoc tests. Cellular experiments were repeated three times

Next, we further investigated whether HDAC inhibitor ITF2357 affected the resistance of mut-KRAS NSCLC cells to Pem by regulating HDAC2/Rad51. First, RT-PCR and Western blot results displayed that versus that in normal human BEAS-2B cells, the expression level of Rad51 was increased in A549 and A549R cells. Relative to A549 cells, A549R cells had notably increased expression level of Rad51 (Fig. 4A, B). Then, we analyzed the correlation between Rad51 and HDAC in TCGA-LUAD through LinkedOmics database, and the results found that Rad51 was positively correlated with HDAC2 (Fig. 4C).

We further explored whether ITF2357 affected the expression of Rad51 by inhibiting HDAC2. It was shown that ITF2357 brought about declines in the expression levels of HDAC2 and Rad51, and oe-Rad51 led to no significant change in terms of the expression level of HDAC2 but increased that of Rad51. Compared with ITF2357 alone, ITF2357 + oe-Rad51 contributed to no significant change in the expression level of HDAC2, but elevated that of Rad51 (Fig. 4D, E). Overall, HDAC inhibitor ITF2357 inhibited the expression of Rad51 by downregulating HDAC2.

We further verified how HDAC inhibitor ITF2357 affected the resistance of A549R cells to Pem through Rad51. Firstly, MTT and Transwell assays following PEM treatment showed that ITF2357 decreased the cell viability, migration and invasion, while oe-Rad51 markedly increased them; compared with ITF2357 alone, ITF2357 + oe-Rad51 notably promoted the cell viability, migration and invasion (Fig. 4F, G). In addition, flow cytometry results revealed that relative to that upon oe-NC, the cell apoptosis in the presence of ITF2357 alone was increased, while that in response to oe-Rad51 was markedly decreased. Compared with ITF2357 alone, ITF2357 + oe-Rad51 contributed to decreased cell apoptosis (Fig. 4H). In conclusion, HDAC inhibitor ITF2357 reduced the resistance of mut-KRAS NSCLC cells to Pem by downregulating Rad51 through HDAC2 inhibition.

### HDAC inhibitor ITF2357 reduced the resistance of mut-KRAS NSCLC cells to Pem by inhibiting HDAC2/miR-130a-3p

**Fig. 5:**
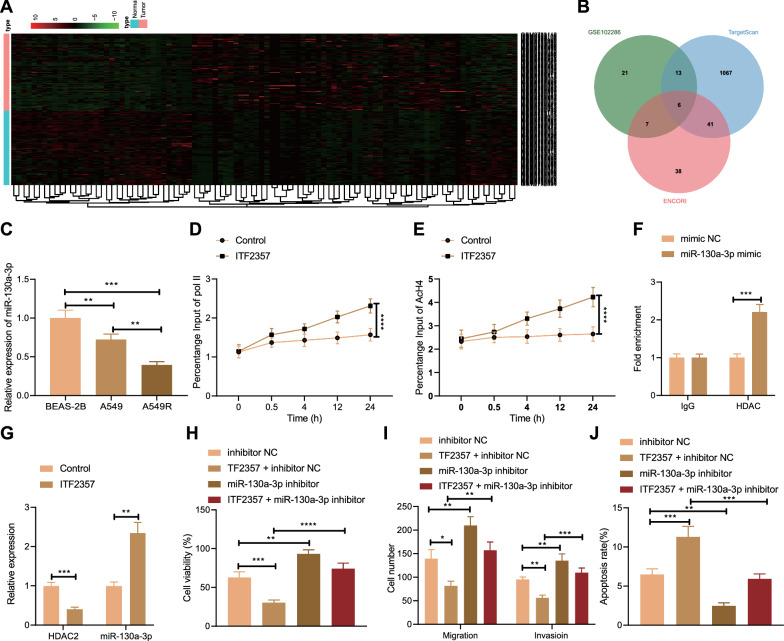
HDAC inhibitor ITF2357 reduces the resistance of mut-KRAS NSCLC cells to Pem by inhibiting HDAC2/miR-130a-3p. **A** Heatmap of differentially expressed genes between control samples and NSCLC samples in GSE102286 (normal = 88, tumor = 91). **B** Venn map shows the overlap of the downregulated miRs in GSE102286 and miRs that target Rad51 as predicted by TargetScan and ENCORI. **C** RT-qPCR was used to detect the gene expression level of miR-130a-3p in cells upon different treatments. **D** ChIP was used to detect the RNA pol II binding in miR-130a-3p promoter region. **E** ChIP was used to detect the binding of AcH4 in miR-130a-3p promoter region. **F** ChIP was used to detect the binding of HDAC2 in miR-130a-3p promoter region. **G** RT-qPCR was used to detect the gene expression levels of HDAC2 and miR-130a-3p. **H** MTT assay was used to detect the viability of cells upon different treatments. **I** Transwell assay was used to detect the migration and invasion of cells upon different treatments. **J** Flow cytometry was used to detect the apoptosis of cells upon different treatments. *p < 0.05. **p < 0.01. ***p < 0.001. ****p < 0.0001. Data between two groups were compared by unpaired t test, and those among multiple groups were compared by one-way ANOVA followed by Tukey’s post hoc tests. Data among multiple groups at different time points were compared by repeated measures ANOVA, followed by Bonferroni post hoc tests. Cellular experiments were repeated three times

Next, we studied whether HDAC inhibitor regulated Rad51 through targeting downstream miRNA. We screened GSE102286 from GEO database (https://www.ncbi.nlm.nih.gov/gds) and obtained 98 differentially expressed miRs (47 were downregulated and 51 were upregulated) (Fig. 5A). Subsequently, the miRs that were downregulated in NSCLC in GSE102286 dataset were intersected with the miRs that target Rad51 as predicted by TargetScan and ENCORI (http://starbase.sysu.edu.cn/index.php) databases, and six miRs were obtained (Fig. 5B). Among the obtained miRs, only miR-130a-3p was reported to be regulated by HDAC2 and downregulated in chemo-resistant NSCLC cells to promote cisplatin resistance [16, 17]. Therefore, we further explored whether HDAC2 affected the resistance of mut-KRAS NSCLC cells to Pem by regulating miR-130a-3p.

First, RT-qPCR results confirmed that relative to that in BEAS-2B cells, miR-130a-3p expression in A549 and A549R cells was markedly diminished; versus that in A549 cells, the expression level of miR-130a-3p in A549R cells was also decreased (Fig. 5C). It was further verified that ITF2357 affected the histone deacetylation of miR-130a-3p promoter. The results of ChIP assay revealed that ITF2357 notably improved the binding of Pol II to miR-130a-3p promoter and enhanced the acetylation of histone H4 (Fig. 5D, E). Subsequently, further ChIP assay found that the amount of miR-130a-3p pulled down by IgG antibody was not different from that by mimic NC, while the amount of miR-130a-3p pulled down by HDAC antibody was higher than that of mimic NC (Fig. 5F), demonstrating that miR-130a-3p can bind to HDAC2. In addition, based on RT-qPCR results, relative to the control cells, ITF2357-treated A549 cells had a marked decrease in HDAC2 level and an increase in miR-130a-3p level (Fig. 5G), indicating that HDAC inhibitor ITF2357 enhanced the expression of miR-130a-3p by inhibiting HDAC2.

Next, we further verified whether HDAC inhibitor ITF2357 affected the resistance of A549R cells to Pem via miR-130a-3p. First of all, MTT and Transwell assays showed that compared with inhibitor NC, ITF2357 led to decreased cell viability, migration and invasion, while miR-130a-3p inhibitor contributed to increased cell viability, migration and invasion. Relative to those upon ITF2357 treatment alone, the cell viability and migratory and invasive potentials of PEM-treated cells in the presence of ITF2357 + miR-130a-3p inhibitor were notably increased (Fig. 5H, I). In addition, based on flow cytometry results, ITF2357 increased the cell apoptosis, while miR-130a-3p inhibitor diminished it. Compared with ITF2357 alone, ITF2357 + miR-130a-3p inhibitor led to markedly decreased apoptosis (Fig. 5J). Taken together, ITF2357 diminished the resistance of mut-KRAS NSCLC cells to Pem by inhibiting HDAC2 and upregulating miR-130a-3p.

### HDAC inhibitor ITF2357 reduced Rad51 expression by inhibiting HDAC2 and upregulating miR-130a-3p

**Fig. 6:**
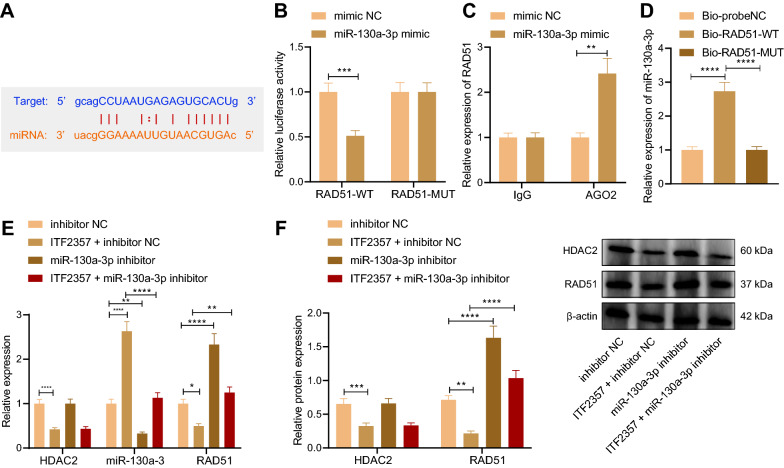
HDAC inhibitor ITF2357 represses Rad51 expression by inhibiting HDAC2 and upregulating miR-130a-3p. **A** The potential binding sites between miR-130a-3p and Rad51 predicted by the Targetscan website. **B** Dual luciferase reporter gene assay to detect the targeting relationship between miR-130a-3p and Rad51. **C** RIP assay to detect the interaction between miR-130a-3p and Rad51. **D** RNA pull-down assay to detect the interaction between miR-130a-3p and Rad51. **E** RT-qPCR to detect the gene expression levels of HDAC2, miR-130a-3p and Rad51. **F** The protein expression levels of HDAC2 and Rad51 in cells upon different treatments were detected by Western blot. **p < 0.01. ***p < 0.001. ****p < 0.0001. Data between two groups were compared by unpaired *t* test, and those among multiple groups by one-way ANOVA, followed by Tukey’s post hoc tests. Cellular experiments were repeated three times

In addition, we further investigated whether HDAC inhibitor ITF2357 regulated miR-130a-3p via HDAC2, thereby affecting the expression of Rad51. The prediction of TargetScan website (http://www.targetscan.org/vert_71/) revealed a specific binding region between miR-130a-3p sequence and Rad51 gene sequence (Fig. 6A). Moreover, dual luciferase gene report assay revealed that in Rad51-mut, no significant change was found in terms of the luciferase activity between mimic NC and miR-130a-3p, while in Rad51-wt, the luciferase activity of miR-130a-3p was lower than that of mimic NC (Fig. 6B), suggesting that miR-130a-3p can target Rad51.

In addition, RIP results demonstrated that Rad51 expression in response to miR-130a-3p was higher than that in the presence of mimic NC (Fig. 6C), suggesting that miR-130a-3p may directly interact with Rad51. Results of RNA pull-down assay then indicated that miR-130a-3p expression in the presence of Bio-Rad51-wt was higher than that upon Bio-probeNC and Bio-Rad51-mut (Fig. 6D). The results of RT-qPCR and Western blot showed that the expression level of miR-130a-3p in response to ITF2357 was increased, while the expression levels of HDAC2 and Rad51 were markedly decreased. Versus ITF2357 alone, ITF2357 + miR-130a-3p inhibitor resulted in a decline in the expression level of miR-130a-3p and an increase in the expression level of Rad51, and no obvious changes in HDAC2 expression (Fig. 6E, F). These results suggest that HDAC inhibitor ITF2357 repressed Rad51 expression by inhibiting HDAC2 and increasing miR-130a-3p.

### HDAC inhibitor ITF2357 reduced the resistance of mut-KRAS NSCLC to Pem via the HDAC2/miR-130a-3p/Rad51 axis

**Fig. 7:**
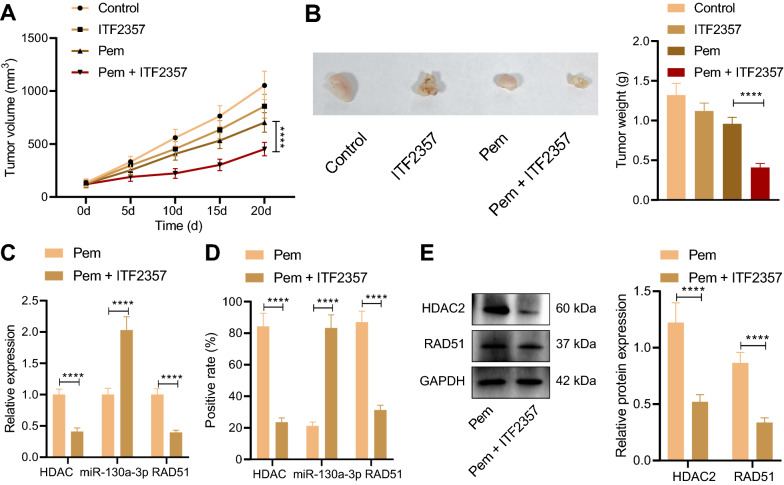
HDAC inhibitor ITF2357 reduces the resistance of mut-KRAS NSCLC to Pem by inhibiting HDAC2/miR-130a-3p/Rad51 in vivo. **A** The changes of tumor size in mice upon different treatments. **B** Tumor weight analysis of mice upon different treatments. **C** RT-qPCR was used to detect the gene expression levels of HDAC2, miR-130a-3p and Rad51 in tumor tissues of mice upon different treatments. **D** Immunohistochemistry and in situ hybridization were used to determine the expression levels of HDAC2, Rad51 and miR-130a-3p in tumor tissues of mice upon different treatments. **E** The protein expression levels of HDAC2 and Rad51 in tumor tissues of mice upon different treatments were detected by Western blot. There were 5 mice in each group. ****p < 0.0001. Data between two groups were compared by unpaired *t* test. Data among multiple groups were compared by one-way ANOVA, followed by Tukey’s post hoc tests. Data among multiple groups at different time points were compared by repeated measures ANOVA, followed by Bonferroni post hoc tests

Next, to further study the inhibitory effect of ITF2357 on the in vivo growth of Pem-resistant mut-KRAS NSCLC, A549R cells were injected subcutaneously into mice to establish tumor-bearing mice model, and ITF2357 alone or Pem in combination were given to monitor tumor growth. The tumor size and weight of mice treated with ITF2357 + Pem were lower than those in mice exposed to Pem alone (Fig. 7A, B). In addition, the tumor tissues were taken out for HE staining, TUNEL and Ki67 immunohistochemistry, as shown in Additional file 1: Fig. S1. Results of HE staining revealed that the tumor cells in the presence of Pem alone were closely arranged, with large nuclei and few cytoplasm, and had obvious cellular atypia, while the tumor cells in the presence of ITF2357 + Pem were relatively reduced and replaced by fibrous tissue Based on the results of TUNEL staining, compared with the Pem alone, ITF2357 + Pem led to decreased number of tumor cells. Moreover, results of immunohistochemistry displayed that the expression level of Ki67 in tumor tissue after treatment with ITF2357 + Pem was lower than that upon Pem treatment alone, demonstrating that the viability ability of tumor cells was down-regulated in response to ITF2357 + Pem. These results suggest that HDAC inhibitor ITF2357 can reduce the resistance of mut-KRAS NSCLC to Pem.

Subsequently, RT-qPCR results showed that relative to Pem alone, Pem + ITF2357 markedly decreased the expression levels of HDAC2 and Rad51 in tumor tissues of mice while increasing that of miR-130a-3p were increased (Fig. 7C). In addition, the results of immunohistochemistry, in situ hybridization and Western blot were consistent with the trends detected in RT-qPCR (Fig. 7D, E). In conclusion, HDAC inhibitor ITF2357 may inhibit Rad51 and reduce the resistance of mut-KRAS NSCLC to Pem by inhibiting HDAC2 and upregulating miR-130a-3p.

## Discussion

Lung cancer is one of the leading causes of cancer death worldwide [18], and almost 80–85% of lung cancers are NSCLC [19]. In spite of the improvement in chemotherapy or targeted therapies, there is still a low survival rate of NSCLC [20]. In the current study, we explored the regulatory mechanism of HDAC inhibitor ITF2357 in the chemo-resistance of mut-KRAS NSCLC to Pem and found that HDAC inhibitor ITF2357 could reduce resistance of mut-KRAS NSCLC to Pem by regulating the miR-130a-3p/HDAC2/Rad51 axis.

It has been well-established that HDACs are enzymes functioning to remove the acetyl groups from hyperacetylated histones [21] and participate in cancer progression via modulation of tumor cell proliferation, differentiation and cell cycle [22]. Recently, an increasing number of studies have unveiled the participation of HDAC2 in the development and chemotherapeutic effect of NSCLC. For instance, HDAC2 could facilitate the migration and invasion of NSCLC cells by increasing fibronectin [23]. Besides, upregulated HDAC2 by FKBP3 was found to contribute to enhanced viability of NSCLC [24]. In addition, overexpressed HDAC2 could lead to reduced cisplatin sensitivity of NSCLC cells induced by valproic acid [8]. Consistent with the previous studies, we found in the present study that HDAC2 expression was upregulated in NSCLC tissues and cells.

Intriguingly, accumulating evidence has further indicated that HDAC inhibitors, such as entinostat, SAHA, trichostatin A and KA2507, could suppress the growth of cancer cells and induce apoptotic cell death in a variety of malignancies including metastatic uveal melanoma, lung cancers, glioblastoma, etc. [25–28]. ITF2357 (givinostat) is a safe and tolerable pan-HDACi with broad anti-inflammatory properties [29], and the anti-tumoral activity of ITF2357 has been reported in various tumors, including NSCLC [7, 30, 31]. In the current study, we illuminated that HDAC inhibitor ITF2357 inhibited the expression of HDAC2, thereby reducing the resistance of mut-KRAS NSCLC cells to Pem. In line with our results, it also has been highlighted that ITF2357 could promote the cytotoxicity of Pem in NSCLC and contribute to increased suppression of xenografted tumor growth in vivo as well as prolonged mice survival [7]. Moreover, a previous study has revealed that ITF2357 could induce the death of ruxolitinib-resistant cells and augment the effect of chemotherapy in B-cell precursor acute lymphoblastic leukemia [32].

Importantly, our molecular mechanism exploration in this study revealed that inhibition of HDAC2 promoted the expression of miR-130a-3p, thereby inhibiting Rad51, which ultimately reduced the resistance of mut-KRAS NSCLC to Pem. To our knowledge, the regulatory relationship between HDAC2 and miR-130a-3p has been rarely reported. There is also a paucity of reports unveiling the interaction between miR-130a-3p and Rad51. In the current study, we discovered a negative correlation between HDAC2 and miR-130a-3p and a positive correlation between Rad51 and HDAC2 in NSCLC based on the results from RT-qPCR and Western blot assay. In consistency with our finding, several previous studies have unveiled the role of Rad51 in NSCLC as an oncogene. For example, upregulation of Rad51c is predictive of poor prognosis of patients with NSCLC and promotes cell resistance to cisplatin as well as radiation in this malignancy [33]. Intriguingly, knockdown of endogenous Rad51 could suppress the growth of the A549 lung cancer cells through accumulation of cells in G1 phase and induction of cell death, thereby serving as an independent prognostic gene in patients with NSCLC [34].

Of note, previous research has also identified miR-130a-3p as an inhibitor of the development of NSCLC. It was demonstrated that miR-130a-3p displayed marked downregulation in patients with NSCLC in comparison to that in healthy individuals [12]. In addition, miR-130a-3p was found to be underexpressed in A549 and H1299 lung cancer cell lines and could suppress the migration of lung cancer cells [35]. Furthermore, Hu et. al. revealed that miR-130a-3p could target SOX4 to augment cisplatin resistance of NSCLC cells and thus enhance efficacy of chemotherapy for NSCLC patients [17].

## Conclusion

**Fig. 8:**
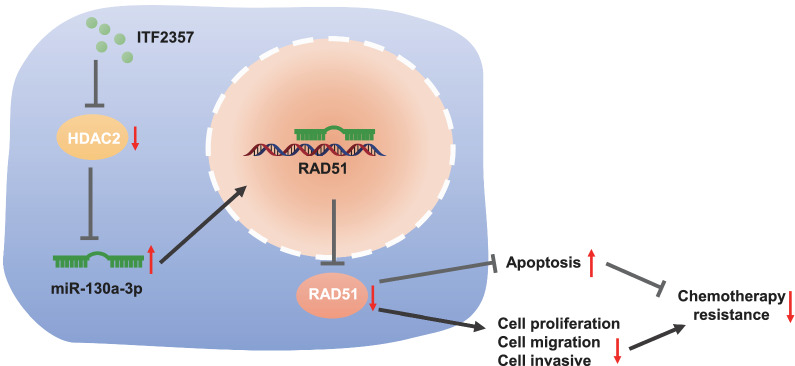
Molecular mechanism graph. HDAC inhibitor ITF2357 elevates the expression of miR-130a-3p by inhibiting HDAC2, which inhibits Rad51, thereby reducing the resistance of mut-KRAS NSCLC to Pem

Taken together, it is concluded in the current study that HDAC inhibitor ITF2357 downregulates HDAC2, and thus increases the expression of miR-130a-3p and decreases the expression of Rad51, diminishing the resistance of mut-KRAS NSCLC to Pem (Fig. 8). This finding may provide a novel direction for control of chemoresistance of mut-KRAS NSCLC. Despite that, the clinical feasibility still warrants further confirmation.

## Supplementary Information

**Additional file 1: Figure S1.** HE staining, TUNEL staining and Ki67 immunohistochemistry were used to detect the tumor histopathology, apoptosis and cell viability in mice upon different treatments. There were 5 mice in each group. **** *p* < 0.0001. Data among multiple groups were compared by one-way ANOVA, followed by Tukey’s post hoc tests.

**Additional file 2: Table S1.** Primer sequences for RT-qPCR. Note: HDAC2, histone deacetylase 2; miR-130a-3p, microRNA-130a-3p; RT-qPCR, reverse transcription-quantitative polymerase chain reaction.

## Data Availability

All data generated or analysed duing the present study are included in this published article.

## Abbreviations

HDAC: Histone deacetylases
NSCLC: Non-small cell lung cancer
Pem: Pemetrexed
HDAC: Histone deacetylase
RT-qPCR: Reverse transcription-quantitative polymerase chain reaction
RIP: Radioimmunoprecipitation
NC: Negative control
ChIP: Chromatin immunoprecipitation
