# Supplementary material for: HDAC inhibitor ITF2357 reduces resistance of mutant-KRAS non-small cell lung cancer to pemetrexed through a HDAC2/miR-130a-3p-dependent mechanism
Source: J Transl Med. 2023 Feb 15;21:125. doi: 10.1186/s12967-023-03973-3 (PMC9930237; doi:10.1186/s12967-023-03973-3)
Supplement: Supplementary file 2 — Additional file 2: Table S1. Primer sequences for RT-qPCR. Note: HDAC2, histone deacetylase 2; miR-130a-3p, microRNA-130a-3p; RT-qPCR, reverse transcription-quantitative polymerase chain reaction. [file 12967_2023_3973_MOESM2_ESM.docx]

**Table S1** Primer sequences for RT-qPCR

| Gene | Sequences |
| --- | --- |
| HDAC2 | Forward: CATGCGGATTCTATGAGGCT |
|  | Reverse: ATGGCGTACAGTCAAGGAGG |
| miR-130a-3p | Forward: GGCAGTGCAATGTTAAAAG |
| Rad51 | Forward: TGGCCCACAACCCATTTCAC |
|  | Reverse: TCAATGTACATGGCCTTTCCTTCAC |
| U6 | Forward: CGCTTCGGCAGCACATATAC |
|  | Reverse: TTCACGAATTTGCGTGTCATC |
| β-actin | Forward: ATCACCATTGGCAATGAGCG |
|  | Reverse: TTGAAGGTAGTTTCGTGGAT |

Note: HDAC2, histone deacetylase 2; miR-130a-3p, microRNA-130a-3p; RT-qPCR, reverse transcription-quantitative polymerase chain reaction
